# Evidence for Pro-Inflammatory Activity of LTα3 on Macrophages: Significance for Experimental Arthritis and for Therapeutic Switching in Rheumatoid Arthritis Patients

**DOI:** 10.3390/ijms26136355

**Published:** 2025-07-01

**Authors:** Ariane Benezech, Jacques-Eric Gottenberg, Yannick Degboé, Andrey Kruglov, Jane Grogan, Fabienne Briand-Mésange, Alain Cantagrel, Adeline Ruyssen-Witrand, Jean-Luc Davignon

**Affiliations:** 1 INSERM U1043, Centre de Physiopathologie de Toulouse Purpan, 31059 Toulouse, France; benezech.a@chu-toulouse.fr (A.B.); yannick.degboe@inserm.fr (Y.D.); fabienne.briand-mesange@inserm.fr (F.B.-M.); alain.cantagrel@wanadoo.fr (A.C.); 2 Service de Rhumatologie, CHU de Strasbourg, 67098 Strasbourg, France; jacques-eric.gottenberg@chru-strasbourg.fr; 3 Centre de Rhumatologie, CHU de Toulouse, 31059 Toulouse, France; ruyssen-witrand.a@chu-toulouse.fr; 4 Deutsches Rheuma-Forschungszentrum, 10117 Berlin, Germany; kruglov@drfz.de; 5 Genentech, San Francisco, CA 94080, USA; jane.grogan@gmail.com; 6 Inserm UMR 1027, CHU Purpan, 31062 Toulouse, France

**Keywords:** soluble lymphotoxin alpha, LTα3, TNF, macrophages, polarization, rheumatoid arthritis, biotherapy, switch, mouse model of arthritis, K/BxN

## Abstract

Lymphotoxin-alpha (LTα3) is a soluble cytokine of the TNF superfamily. Its role in inflammation and arthritis is not well known. Macrophages are important in K/BxN Serum-Transfer Arthritis (STA) and rheumatoid arthritis (RA). Anti-TNF monoclonal antibodies as well as etanercept (ETA), a soluble TNF receptor II that also neutralizes LTα3, are efficient in the treatment of RA. Objectives: To evaluate the role of LTα3 in macrophage phenotypes and in arthritis. Methods: Macrophages were cultured in the presence of recombinant LTα3, and their phenotypes were studied. The clinical effect of blocking LTα3 in STA was evaluated, as well as the effect of switching from anti-TNF monoclonal antibodies to etanercept in the “ROC” register of RA patients. Results: We showed that recombinant LTα3 was capable of directing mouse and human macrophages towards a pro-inflammatory “M1” phenotype. In K/BxN STA, ETA decreased clinical score and joint swelling. Anti-LTα3 reduced arthritis only in TNF-KO mice, indicating that the effect of LTα3 was visible in the absence of TNF. The “ROC” register indicated that switching anti-TNF mAb to ETA did not induce clinical and biological improvement in RA. Conclusion: We show a pro-inflammatory role for LTα3 in murine and human macrophages. The neutralization of both TNF and LTα3 is not beneficial in the treatment of RA.

## 1. Introduction

Rheumatoid arthritis (RA) is an autoimmune disease whose development and pathology depend on pro-inflammatory cytokines [1]. Anti-TNF and anti-IL-6 biologics have revolutionized RA treatment [2,3]. Other types of biologics that target T- and B-cells contribute also to blocking the autoimmune process and to reducing inflammation [4]. A proportion of patients do not respond to the first biologic that is prescribed and require a switch to drugs that target other components of the immune system [5]. Gottenberg et al. reported in 2016 [5] that RA patients who switch from a TNF inhibitor to any other family of bDMARDs (biological Disease-Modifying AntiRheumatic Drugs) have the best therapeutic outcomes. To obtain this finding, the authors designed a randomized clinical trial called Rotation Of Change “ROC” (clinicaltrials.gov Identifier: NCT01000441).

Lymphotoxin-alpha (LTα) is a cytokine that belongs to the TNF superfamily [6]. There are several isoforms of LT, depending on the trimeric combinations of α and β chains involved in T-cell differentiation, as well as the maturation of lymphoid organs. It has been suggested that LTα3 possesses pro-inflammatory properties [7]. Etanercept (ETA) is a TNF inhibitor constituted of soluble TNF-RII that binds TNF as well as LTα3 [8]. It is widely used in the treatment of RA, but whether its impact on RA relies also on its binding to LTα3 is not known. ETA has been shown to reduce expression of TNF and LTα3 in the synovia of RA patients [9]. LTα3 has been shown to induce pro-inflammatory cytokines in RA synoviocytes [10,11], and to increase the adhesiveness of T-cells [12]; however, LTα3 and TNF possess non-redundant functions in mice with regard to immunity [13].

Targeting surface LTα3 with a surrogate monoclonal antibody was shown to reduce arthritis in the CIA model by the authors of [14]. Efficacy in the murine model depended on its depleting activity on pathogenic T-cells. Based on that study, a role for LTα3 as a target in RA in mice has been suggested, and its effects on RA synovial fibroblasts were considered [13,15]. However, a phase 2 randomized controlled trial found that pateclizumab, a human anti-LTα3 mAb, did not improve clinical RA symptoms when compared with placebo, despite demonstrating clear pharmacodynamic effects [16].

Polarization of macrophages is a marker of inflammation in RA [17]. Anti-TNF treatment limits the pro-inflammatory cytokine and the surface phenotype, and induces a switch to an alternative polarization state [17]. Several models of arthritis have been developed that mimic RA and other inflammatory arthritides [18]. K/BxN mice express both the T-cell receptor (TCR) transgene KRN and the MHC class II molecule A(g7), and develop autoantibodies recognizing glucose-6-phosphate isomerase (GPI), inducing spontaneous severe arthritis. The transfer of serum from these mice induces arthritis (Serum-Transfer Arthritis (STA)) that reaches a peak around 10 days post-injection and resolves in 3 weeks [19]. This model bypasses the T-cell activation step and is dependent on macrophages and neutrophils [20,21].

In this study, given the importance of macrophages in RA, we assessed the role of recombinant LTα3 on the polarization of mouse and human macrophages. We demonstrated that LTα3 induced pro-inflammatory cytokines that modified the phenotype of macrophages towards a pro-inflammatory polarization state. We further evaluated the role of LTα3 on macrophages in vivo using the K/BxN STA model [22,23,24] and showed that LTα3 has a critical role in that model.

Finally, we took a translational approach using the “ROC” register to evaluate whether LTα3 had a role in established RA by assessing the outcome of a therapeutic switch from anti-TNF Ab to ETA, i.e., comparing neutralization of TNF alone with neutralization of TNF + LTα3.

## 2. Results

### 2.1. Induction of CD40 in Mouse Macrophages by LTα3

**Figure 1:**
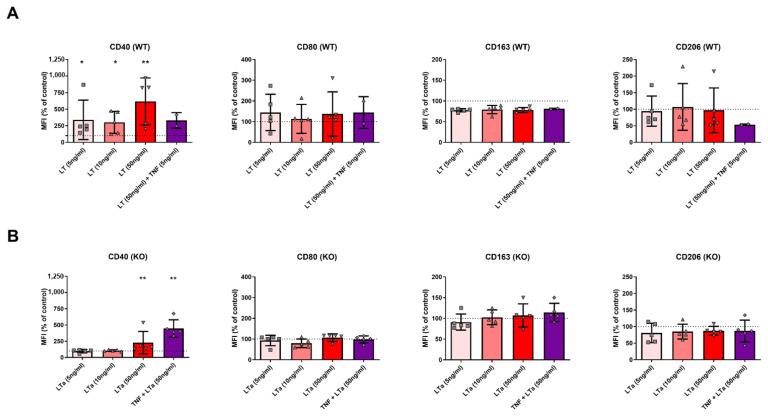
Induction of CD40 by LTα3 in mouse macrophages. Bone marrow was collected from TNF+/+ (WT) mice and cultured with M-CSF. LTα3 was added at the indicated concentrations for the last 24 h (**A**). Similarly, bone marrow was collected from TNFko/ko (KO) mice and cultured with M-CSF. LTα3 was added at the indicated concentrations for the last 24 h (**B**). Expression of surface markers on macrophages was analyzed by flow cytometry on day 5 after initiation of the culture. Each symbol represents a biological replicate of a macrophage population isolated from a single mouse. Statistics were calculated with reference to 100% standardization values. * *p* < 0.05, ** *p* < 0.01.

In order to delineate the phenotype of macrophages in the presence of LTα3 and in the absence of TNF, BMDMs were prepared in the presence of M-CSF, which does not induce TNF secretion, and then incubated with LTα3 at different concentrations. Figure 1A shows that only CD40, a pro-inflammatory marker of macrophages, was significantly increased; notably, at all concentrations of LTα3. Adding TNF reversed the CD40 phenotype induced by LTα3 alone in TNF+/+ mice, although samples from only two individual mice were analyzed. However, CD80 was not increased. As for the expression of the pro-resolutive CD163 and CD206, these were not significantly modified.

To verify the relevance of LTα3 in the absence of TNF, we carried out the same experiments using BMDMs from TNFko/ko mice. Figure 1B shows that in the M-CSF-treated BMDMs from TNFko/ko mice, CD40 expression was induced in the presence of LTα3, thus confirming the effectiveness of LTα3 in the absence of TNF, similarly to TNF+/+ mice. In TNFko/ko mice, adding TNF induced a significant increase in CD40 expression, although the other markers were not modified. MFI values for CD163 were very low and appeared scattered. TNF alone was not evaluated in this study. Figure S1 shows MFI ratios used for standardization and determination of percentages of control. Figure S2 shows the gating strategy and original histograms of monocytes labeling.

### 2.2. Induction of Mouse Pro-Inflammatory Cytokines by LTα3

**Figure 2:**
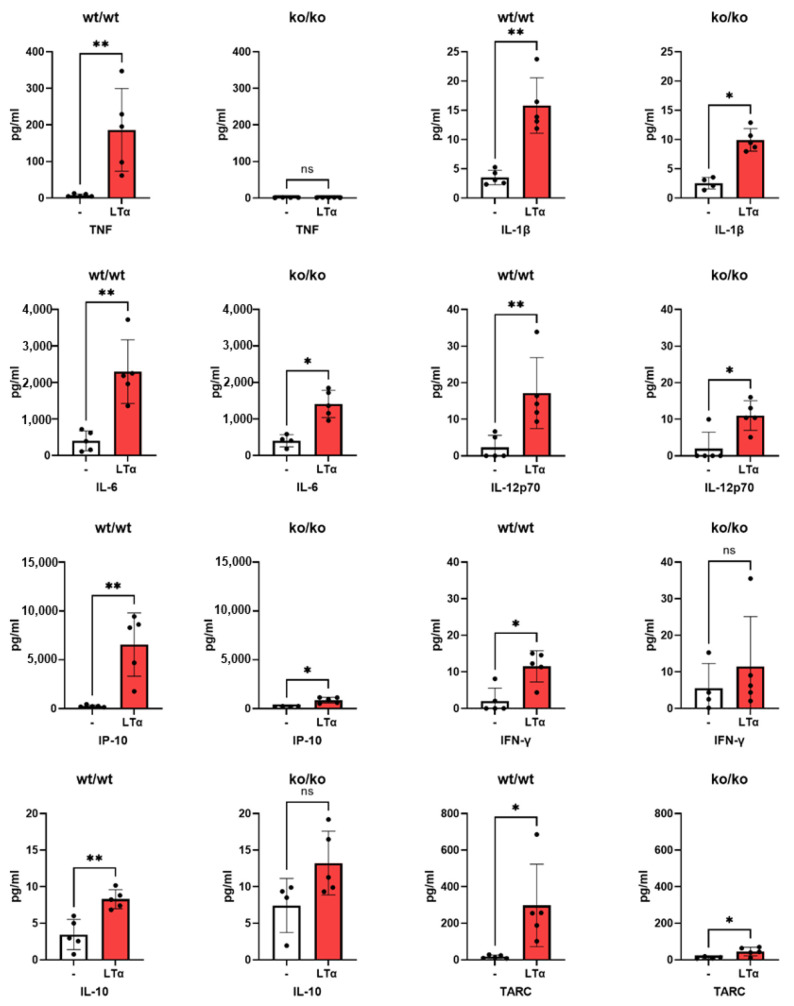
Induction of pro-inflammatory cytokines by mouse macrophages in the presence of LTα3. Bone marrow was collected from TNF+/+ (wt/wt) mice and cultured with M-CSF. LTα3 was added for the last 24 h. Similarly, bone marrow was collected from TNF−/− (ko/ko) mice and cultured with M-CSF. LTα3 was added for the last 24 h. Expression of surface markers on macrophages was analyzed by flow cytometry on day 5 after initiation of the culture. Each symbol represents a biological replicate of a macrophage population isolated from a single mouse. * *p* < 0.05, ** *p* < 0.01.

Using the same culture protocol, supernatants were tested for the production of various cytokines which could represent inflammation or control thereof. LTα3 induced significant amounts of TNF, IL-1, IFN-γ, IL-12, and IL-6 in macrophages from TNF+/+ mice (Figure 2). As a control, BMDMs from TNF ko/ko mice did not produce TNF, as expected, and their production of IL-1 (*p* = 0.033), IP-10 (*p* < 0.01), and TARC (*p* < 0.01) was lower than that of +/+ mice, suggesting that their induction by LTα3 was at least in part dependent on the presence of TNF. Production of IL-10 by BMDMs from TNFko/ko mice was higher than that of +/+ but significance was low (*p* = 0.048). It is of note that some of the cytokines were below the detection range.

### 2.3. Role of LTα3 in K/BxN STA Arthritis

In a first approach to assess the role of LTα3 in the effector phase of arthritis, C57BL/6 mice were treated with antibody specific for LTα3 or with ETA that neutralizes mouse TNF, as well as LTα3. Arthritis was then induced using the classical K/BxN serum-transfer model. This model is dependent on IL-1 but only partially on TNF [25,26].

**Figure 3:**
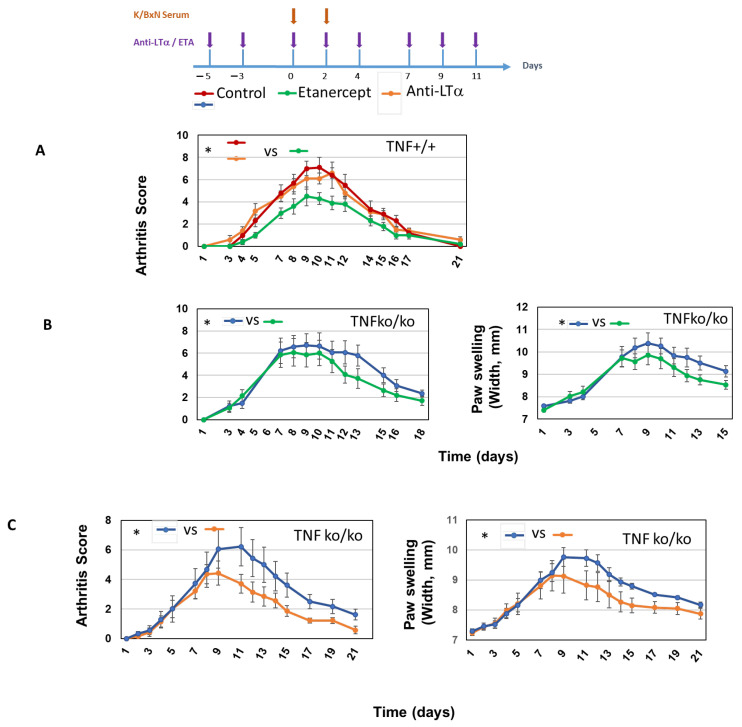
Decreased arthritis in mice through neutralization of LTα3 in the absence of TNF. C57BL/6-TNF+/+ mice (*n* = 6–8) were injected IP with anti-LTα3, ETA, or isotype control as indicated, then treated IP with K/BxN serum according to the indicated schedule. Individual mice were scored in a blinded fashion for arthritis. Mean arthritis scores are represented (**A**). C57BL/6-TNFko/ko mice were injected IP with ETA or isotype control (**B**) and anti-LTα3 antibody or isotype control (**C**), as indicated, then treated IP with K/BxN serum according to the indicated schedule. Individual mice were scored in a blinded fashion for arthritis. Mean arthritis scores are represented. Ankles were measured using a caliper. Mean arthritis scores and mean measurements of ankles (Right + Left) are represented (**B**). Data represent mean + SEM. The statistical significance of clinical scores and ankle measurements for the whole course of arthritis was calculated by repeated measurements of two-way ANOVA tests. * *p* < 0.05.

Figure 3A shows that anti-LTα3 alone did not change the course of the disease in TNF+/+ mice, as measured by the arthritis clinical score. This indicated that LTα3 alone has little role in the effector phase of arthritis in this model. However, ETA treatment did result in lower arthritis scores, indicating that either TNF alone, as already reported [25,26], or TNF in association with LTα3 was involved in this model. We took advantage of a previous report of TNFko/ko mice exhibiting significant K/BxN arthritis that was only partially dependent on TNF [25]. We thus used TNFko/ko mice in order to assess the role of LTα3 in the absence of TNF. Treatment of TNFko/ko with ETA resulted in significantly reduced symptoms of the disease as assessed by the arthritis score and the swelling of ankles, as shown in Figure 3B. This suggested that LTα3 was involved in arthritis in mice that did not express TNF. To definitely prove the role of LTα3, we treated TNFko/ko mice with an anti-LTα3 antibody and assessed the clinical score and swelling. Figure 3C shows that neutralizing LTα3 in arthritis in the absence of TNF alleviated the intensity of clinical symptoms in a statistically significant fashion.

### 2.4. Induction of Human M1 Phenotype and Decrease in M2 Markers by LTα3 in the Absence of TNF

**Figure 4:**
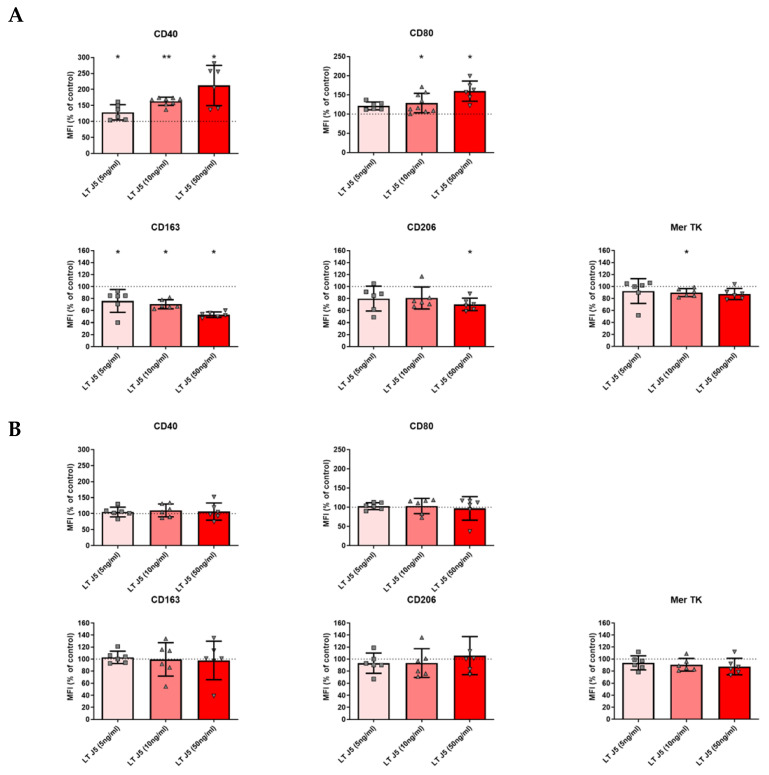
Induction of pro-inflammatory phenotype by LTα3 in human macrophages. Monocytes were purified and cultured with M-CSF in the absence (**A**) or presence (**B**) of IFN-γ + LPS (I + L) for the last 24 h. LTα3 was added at the indicated concentrations for the last 24 h. Expression of surface markers on macrophages was analyzed by flow cytometry on day 5 after initiation of the culture. Each symbol represents a separate blood donor. Statistics were calculated with reference to the 100% standardization values. * *p* < 0.05, ** *p* < 0.01.

Experiments were performed on macrophages from healthy blood donors. Purified monocytes were cultured in the presence of M-CSF and LTα3. Markers of M1 and M2 polarization were evaluated. As shown in Figure 4A, LTα3 was able to increase the expression of CD40 and CD80 as well as decrease the expression of CD163 and CD206. However, in the presence of LPS + IFN-γ, which induces TNF, no modifications were observed (Figure 4B). Thus, LTα3 switched macrophages towards an M1-like surface phenotype when TNF was absent.

**Figure 5:**
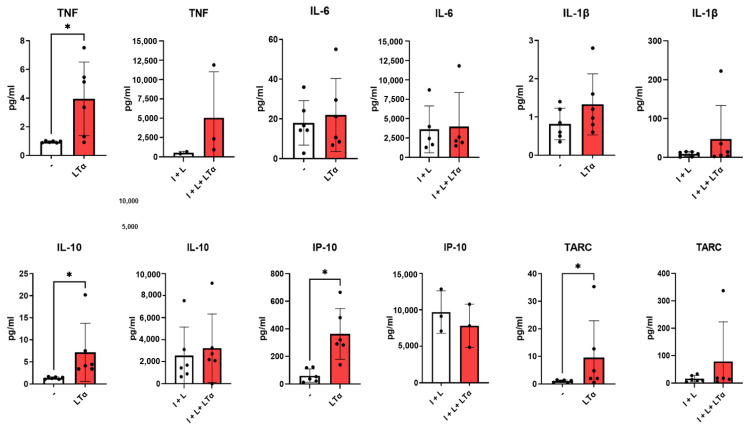
Induction of pro-inflammatory cytokines by human macrophages in the presence of LTα3. Monocytes were purified and cultured with M-CSF in the absence or presence of LTα3 (50 ng/mL). Supernatants of cultures in the absence or presence of IFN-γ + LPS (I + L) for the last 24 h were tested for cytokine production using a CBA. Each symbol represents a separate blood donor. * *p* < 0.05.

Cytokines were measured in the supernatants (Figure 5). LTα3 was found to induce the production of TNF, although at very low levels. LTα3 also increased the secretion of TARC and IP10. As a retro-control of inflammation, IL-10 was induced by LTα3 as expected [17]. In the presence of IFN-γ + LPS, which induces TNF, no such modifications were observed when LTα3 was added, except with regard to TNF production; however, in this case, the number of samples was too small to reach significance. Thus, LTα3 was capable of inducing pro-inflammatory phenotypes in macrophages in the absence of TNF, as well as retro-control of inflammation. All comparisons between control and I + L on the one hand and LTa and I + L + LTa on the other hand were highly significant (<0.01), as expected from the strong increase in cytokine production (from 10 times to 1000 times) in the presence of IFN-γ + LPS. Unlike with mice, IL-1β and IL-6 were not induced (Figure 5).

### 2.5. RA Patients from the “ROC” Registry Did Not Benefit from Switch to ETA

The “ROC” trial, as presented in Table 1, included patients who had inadequate response to a first anti-TNF therapy and were randomized to a second anti-TNF or a bDMARD with another mode of action (tocilizumab, abatacept, or rituximab, according to physician choice). We took advantage of this study to assess cycling in the anti-TNF class that included neutralization of LTα3, i.e., ETA. We assumed that if LTα3 played an important role in the clinical course of RA patients, cycling from anti-TNF monoclonal antibody to ETA, which neutralizes both TNF and LTα3, would be beneficial. Table 2 shows the differences in DAS28 after 6 months of cycling of drugs, as compared to the sequence anti-TNF mAb to ETA. Comparisons of anti-TNF mAb to ETA switch with cycling from ETA to monoclonal antibody and with cycling from mAb to another mAb indicated a lack of benefit in both cases (*p* = 0.289 and 0.178, respectively). However, cycling from ETA or an anti-TNF mAb to another class (CTLA4-Ig abatacept of IL-6R Tocilizumab) was beneficial (*p* = 0.0017), as already reported by Gottenberg et al. [5].

## 3. Discussion

In this study, we identified a pro-inflammatory role of LTα3 in murine and human macrophage polarization. We obtained evidence that LTα3 can modulate K/BxN STA in the absence of TNF. However, we found that this was not the case in RA, as demonstrated in a trial (“ROC”) based on anti-TNF cycling from anti-TNF monoclonal antibody to ETA. This translational approach proved to be helpful in underlining the disparity between the mouse-model and human data.

Although several other cell populations, such as neutrophils and FLS, are involved in the effector phase of arthritis [27,28], macrophages have prominent roles in both experimental arthritis and in RA [22,23,24]. Our findings on macrophages thus have implications for RA pathophysiology.

Macrophages were submitted to stimuli that were identical and not subject to any previous cytokine encounter. Our data suggest that LTα3 may orientate macrophages towards inflammation in K/BxN STA and RA, adding to our understanding of the pathophysiological role of macrophages.

Surface phenotypes of human macrophages tended to be of the M1 profile because, on the one hand, CD40 and CD80 were induced, and, on the other hand, CD163 and CD206 were decreased. Cytokine measurements confirmed such polarization. In mice, cytokines and surface markers did not match exactly those of humans but were also modified towards a pro-inflammatory phenotype, although only CD40 was induced at all LTα3 concentrations.

Because TNF exhibits such powerful activity, the pro-inflammatory role of LTα3 may be masked in some instances when TNF is present. However, the influence of TNF over LTα3 was seen only with CD40 in macrophages from TNFko/ko. We did not investigate the comparative roles of TNF and LTα3 with regard to their capacity to induce cytokine production. However, in TNF+/+ mice, LTα3 induced TNF production, as reflected in a higher production of IP-10 and TARC compared to TNFko/ko mice. Thus LTα3 and TNF can induce pro-inflammatory conditions both separately and mutually. However, LTα3 in the absence of TNF could induce IL-10 production as a retro-control, as observed in TNFko/ko mice.

In humans, in the presence of TNF (i.e., IFN-γ + LPS), the effect of LTα3 was not visible over TNF. This may be explained by the simultaneous production of multiple pro-inflammatory cytokines.

Some notable differences were observed between the roles played by LTα3 in mouse and human macrophages. First of all, LTα3 did not induce TNF in human macrophages whereas higher amounts were induced in murine counterparts, suggesting that neutralizing LTα3 alone in mice may dampen TNF concentrations as well. However, because TNF is not the main actor in STA [25], there were no consequences of anti-LTα3 treatment, as anti-LTα3 alone did not inhibit arthritis in +/+ mice (Figure 3A). However, LTα3 induced the two pro-inflammatory cytokines IL-6 and IL-1β in murine but not human macrophages. Although IL-1β is essential in STA, IL-6 is not [25]. Thus, in the absence of TNF, LTα3 has some significance in the development of STA. Of note, IFN-γ, IL-10, IP-10, and TARC induction by LTα3 were dependent on TNF, as evidenced by the use of TNFko/ko mice, suggesting a role of TNF in response to LTα3. Neutralization of LTα3 has consistently been shown to produce no effects in STA in +/+ mice or in RA (this present study and [16]). This is in accordance with the normal development of STA in LTα-ko mice [25]. Neutralization of LTα3 only ameliorated STA when TNF was not present (Figure 3C). This highlights different mechanisms of STA and RA.

The anti-LTα3 mAb used by Chiang et al. [14] in CIA targeted T-cells that have no relevance in K/BxN STA, as their action is bypassed by anti-GPI antibodies present in the transferred serum [29]. In our present study, we used a mutated mAb (LTα.Fc-MT) that blocked LTα3 trimers but did not induce cytotoxicity or phagocytosis thanks to a mutation in its Fc portion that prevented binding to Fc-receptors and thus specifically inhibited the action of soluble LTα3 [14].

K/BxN has been used for decades as a model of RA. Although some of its features have been observed to be congruent with RA, differences in the pathophysiology of STA and RA have been described [20,21], especially with regard to biological features such as rheumatoid factor and anti-citrullinated protein antibodies (ACPAs) that are present in RA but not in K/BxN.

In our study, even when TNF was neutralized together with LTα3, such as in the presence of ETA, there was no amelioration of the disease, as concluded from the ROC register. The reason may be due to the pathophysiology of RA which, contrary to STA, develops over a long period of and involves T-cell autoimmunity followed by the occurrence of autoantibodies.

The pro-inflammatory properties of LTα3 have been reported previously, but a direct in vivo role in the effector phase of arthritis has not been previously demonstrated. Calmon-Hamaty et al. reported that LTα3 induced the proliferation of RA FLS, and that it was dependent on erk, p38, and PI3K [10]. The involvement of NF-kB was also suggested in [12]. Induction of IL-6, IL-8, and MMP3 by LTα3 has also been reported in RA FLS [15]. In this present study, induction of IL-6 was observed in mouse macrophages as opposed to human macrophages. However, IL-10, IP-10, and TARC were induced by LTα3 in human macrophages. This was observed in mouse macrophages as well, and suggests that LTα3 could contribute to some extent to the migration of cells to inflammatory sites of pannus.

Gottenberg et al. [5] showed that, in the treatment of RA, replacing anti-TNF antibody with another class of bDMARD produced a better EULAR response, in comparison to patients who had anti-TNF cycling. In this study, we found that cycling to ETA that neutralized LTα3 as well did not improve the therapeutic response in patients who did not respond to anti-TNF. This is in contrast with our data for mice which showed that neutralizing both TNF and LTα3 with ETA, or neutralizing LTα3 in TNFko/ko mice, was effective in reducing clinical symptoms of arthritis. Beyond obvious differences in the mechanisms of K/BxN STA and RA [20,21], it should be emphasized that mice with STA did respond, although weakly, to anti-TNF alone [26], but not to anti-LTα3 alone (This present study, Figure 2), thus pointing to a minor role of LTα3 in this model. In RA, biologics are administered at a phase that is beyond the initiation of the disease. We can hypothesize that the role of LTα3 may be confined to the early phase of RA, possibly when T-cells initiate the autoimmune process, as demonstrated in the murine model of CIA [14].

In conclusion, we here demonstrate the pro-inflammatory role of LTα3 in a STA model of mice. However, our translational approach shows that, in RA, there is no benefit from a switch to ETA, in comparison to any other anti-TNF. Our study underlines discrepancies that can occur between mouse models and human disease. Thus, our data are in accordance with a previous report that showed that pateclizumab, an anti-LTα3 mAb, had no influence on the course of RA [16]. Our study shows, nevertheless, that although neutralizing LTα3 is not a therapeutic option in established RA, it does induce pro-inflammatory features in human macrophages. This may open new options in reducing inflammation in other pathologies.

## 4. Materials and Methods

### 4.1. Ethics Approval of the Procedure

Human: Use of blood donors’ samples was possible through the agreement with “EFS” (French Blood Establishment) number 21PLER2021-010-AV01.

Mice: Experiments were performed under the protocol CEEA-122 2014-62, authorized by the “Comité d’éthique en matière d’expérimentation animale CEEA122-US006/CREFRE.

### 4.2. Generation of Human Macrophages

Buffy coat was obtained from healthy donors in the Etablissement Français du sang (Toulouse, France) after written consent. Peripheral blood mononuclear cells (PBMCs) were isolated on Pancoll (Pan Biotech, Aidenbach, Germany). CD14+ monocytes were purified by positive magnetic sorting (Invitrogen Magnisort Human CD14, Waltham, MA, USA) as in Degboé et al. [17]. From the PBMCs obtained, monocytes were isolated using a positive selection kit (Invitrogen MagniSort Human CD14). PBMCs were incubated for 10 min at room temperature with an anti-CD14 antibody coupled to Biotin (concentration of 20 μL per 15 million cells). After washing, Streptavidin-coated magnetic beads were added (30 μL per 15 million cells, 10 min incubation at room temperature). The tube was placed on a magnet for 5 min; then, the unbound fraction was discarded. Three washes were performed with purification buffer. Monocytes were then diluted in culture medium to a concentration of 1 million cells per ml.

Monocytes were cultured throughout at 37 °C/5% CO_2_ in RPMI medium 1640 + Glutamax (Gibco ThermoFisher Scientific, Waltham, MA, USA), supplemented with 10% fetal calf serum (Gibco), and Penicillin G (100 µg/mL) and Streptomycin (100 U/mL) (Invivogen, Toulouse, France).

Monocytes (1 × 10^6^/mL) were differentiated into macrophages using two conditions: In the first condition, monocytes were cultured in the presence of recombinant M-CSF (50 ng/mL; BioLegend, San Diego, CA, USA) for 6 days. In the second condition, “M(M-CSF+LPS/IFN)” macrophages were obtained after 5 days with M-CSF (50 ng/mL, Biolegend) and 24 h with *Escherichia coli* lipopolysaccharides (LPS) (20 ng/mL Sigma Aldrich, St. Louis, MO, USA) and interferon gamma (IFN-γ) (25 ng/mL, Peprotech, ThermoFisher Scientific, Waltham, MA, USA).

Human recombinant Lymphotoxin alpha (LTα3) (5, 10 or 50 ng/mL; eBiosciences, ThermoFisher Scientific, Waltham, MA, USA) was added for the last 24 h of culture.

### 4.3. Mouse Cells

Mice were housed in a specific pathogen-free environment and cared for in accordance with European institutional guidelines (http://eur-lex.europa.eu, accessed on 24 June 2023). TNFko/ko mice originally developed by Marino et al. [30] were obtained from Bernard Ryffel (CNRS Orléans, Orléans, France).

Bone marrow cells were collected from mice. Cells were differentiated under two conditions: recombinant murine M-CSF (50 ng/mL; Biolegend) alone for 6 days; or with *Escherichia coli* lipopolysaccharides (LPS) (Sigma Aldrich, 20 ng/mL) plus recombinant murine interferon gamma (IFN) (Peprotech, 25 ng/mL) for the last 24 h.

Cells were cultured at 37 °C/5% CO_2_ in RPMI medium 1640 + Glutamax (Gibco), supplemented with 10% fetal calf serum (Gibco), and Penicillin G (100 µg/mL) and Streptomycin (100 U/mL) (Invivogen).

Murine recombinant Lymphotoxin alpha (LTα3) (R&D Systems) and/or recombinant human TNF (5 ng/mL; Peprotech) were added on D5 of culture.

Human recombinant Lymphotoxin alpha (LTα3) (5, 10, or 50 ng/mL; eBiosciences) was added for the last 24 h of culture.

### 4.4. Flow Cytometry Analysis

We assessed the effects of LTα3 on macrophages by flow cytometric analysis of membrane markers. Before labeling, macrophages were blocked with a Fc receptor blocking solution (FcR blocking reagent, mouse or human, Miltenyi Biotec, Bergisch Gladbach, Germany). Surface staining on human cells was performed using the following murine anti-human antibodies: CD40 APC/Cy7 (clone 5C3, BioLegend), CD80 BV421 (clone 2D10, BioLegend), CD206 AF488 (clone 15-2, BioLegend), MER proto-oncogene tyrosine kinase (MerTK) PE (clone 125518, R&D systems, Minneapolis, MN, USA), and CD163 FITC (clone GHI/61.1, Miltenyi).

Surface staining on murine cells was performed using rat and hamster anti-human antibodies: CD40 APC (Rat, clone 3/23, Biolegend), CD80 FITC (Armenian hamster, clone 16-10A1, Biolegend), CD163 PE (Rat, clone TNKUPJ, eBioscience), CD206 AF488 (Rat, clone C068C2, Biolegend), and F4/80 PerCP (rat, clone BM8, Biolegend). Only F4/80 positive cells were used for flow cytometric analysis of membrane markers.

We evaluated geometric mean fluorescence intensity (MFI). Fluorescence levels were expressed as a ratio (specific labeling/corresponding isotype), which was standardized with the corresponding control condition.

Cells were analyzed on a MACSQuant 10 (Miltenyi Biotec). Data analysis was performed using FlowJo vX.0.7.

### 4.5. Cytokine Measurements

Culture supernatants were collected and stored at −80 °C until analysis. Concentrations of IL-12p70, TNF-α, IL-6, IL-10, IL-1β, TARC, IL-1RA, IL-12p40, IL-23, and IP-10 were determined simultaneously using bead-based immunoassay (LEGENDplex Mouse M1/M2 Macrophage panel, 740509, Biolegend). Data acquisition was performed on a MACSQuant 10 (Miltenyi Biotec). Data were analyzed using LEGENDplex v8.0.

### 4.6. K/BxN Serum-Transferred Arthritis

Arthritis was induced by I.P. injection on day 1 and day 3 of 200 µL of serum obtained from K/BxN mice of both sexes. Etanercept, a soluble human TNF-RII receptor, was used to block TNF and LTα3. A specific anti-LTα3 antibody that is mutated in the Fc portion was described by Chiang et al. [14]. IgG1 immunoglobulin was used as an isotype control [14].

### 4.7. Patients

The ROC trial was a 52-week, pragmatic, multicenter, open, parallel-group, randomized clinical trial with a superiority design. Originally, patients with insufficient response to an anti-TNF drug were randomly assigned in a 1:1 ratio to receive a non–TNF targeted biologic or a second anti-TNF agent. Patients were recruited from December 2009 to August 2012. In this current study, patients receiving anti-TNF monoclonal antibodies and cycled to etanercept were compared.

### 4.8. Statistical Analysis

We compared membrane marker expression on human cells using a Wilcoxon matched pairs test. Mouse samples were compared using the Mann–Whitney test in GraphPad Prism9. LEGENDplex results were compared using the Mann–Whitney test. A *p* value < 0.05 was considered to be significant, with *** *p* < 0.001, ** *p* < 0.01, and * *p* < 0.05.

Data from in vivo arthritis experiments were analyzed with repeated-measurements two-way ANOVA testing. Data were represented as mean ± SEM, and *p* < 0.05 (two-tailed) was considered to be statistically significant.

## 5. Conclusions

In this study, we demonstrate the pro-inflammatory role of LTα3 in a STA model of mice. However, our translational approach shows that, in RA, there is no benefit from a switch to ETA, compared to any other anti-TNF. Our study underlines discrepancies that can occur between mouse models and human disease. Thus, our data are in accordance with a previous report that showed that pateclizumab, an anti-LTα3 mAb, had no influence on the course of RA [16]. Our study shows, nevertheless, that although neutralizing LTα3 is not a therapeutic option in established RA, it does induce pro-inflammatory features in human macrophages. This may open new options in reducing inflammation in other pathologies.

## Figures and Tables

**Table 1:** Characteristics of ROC patients at inclusion. IQR: Interquartile Range, ESR: Erythrocyte Sedimentation Rate, CRP: C-Reactive Protein, DAS28: Disease Activity Score based on 28 joints.

**Baseline Characteristics of Patients**	
*n*	
Women, *n* (%)	289
Age, Mean (SD), y	240 (83)
Disease Duration, Median (IQR), y	56.5 (48.5–65.3)
Anti-CCP positive, *n* (%)	225/276 (81.5)
Rheumatoid Factor positive, *n* (%)	231/285 (81)
**No. of Joints (28), Median (IQR)**	
Tender	7 (4–11)
Swollen	5 (3–7)
ESR, Median (IQR), mm	24 (11-44)
CRP Level, Median (IQR), mg/L	8 (4–22.2)
DAS28, Mean (SD)	5 (4.3–5.9)
**Concomitant Treatment with a synthetic DMARD, *n* (%)**	
**First anti-TNF prescribed, *n* (%)**	
Etanercept	155 (53.6)
Adalimumab	85 (29.4)
Infliximab	41 (14.2)
Certolizumab pegol	5 (1.7)
Golimumab	3 (1.0)
**Sequence, *n* (%)**	
etanercept : : other class	81 (28.0)
anti-TNF : : other class	66 (22.8)
etanercept : : other anti-TNF	74 (25.6)
anti-TNF : : etanercept	53 (18.3)
anti-TNF : : other anti-TNF	15 (5.2)

**Table 2:** Summary of cycling of biotherapy.

Sequence *n* (%)		Δ DAS28 (6-Months Minus Inclusion)	*p*
Anti-TNF mAb : : Etanercept	53 (18.3)	−1.1 (−1.9–−0.2)	-
Etanercept : : Anti-TNF mAb	74 (26.6)	−1.2 (−1.9–−0.5)	0.289
Anti-TNF mAb : : Other anti-TNF mAb	15 (5.2)	−1.1 (−1.8–0.4)	0.178
Anti-TNF : : Other class	66 (22.8)	−1.9 (−2.9–−1.0)	0.0017

## Data Availability

Data contained within the article.

## Supplementary Materials

The following supporting information can be downloaded at: https://www.mdpi.com/article/10.3390/ijms26136355/s1.
