# Supplementary material for: Evidence for Pro-Inflammatory Activity of LTα3 on Macrophages: Significance for Experimental Arthritis and for Therapeutic Switching in Rheumatoid Arthritis Patients
Source: Int J Mol Sci. 2025 Jul 1;26(13):6355. doi: 10.3390/ijms26136355 (PMC12250361; doi:10.3390/ijms26136355)

Supplementary Figure S1: MFI ratios used for determination of percentages of control.
* p < 0.05.

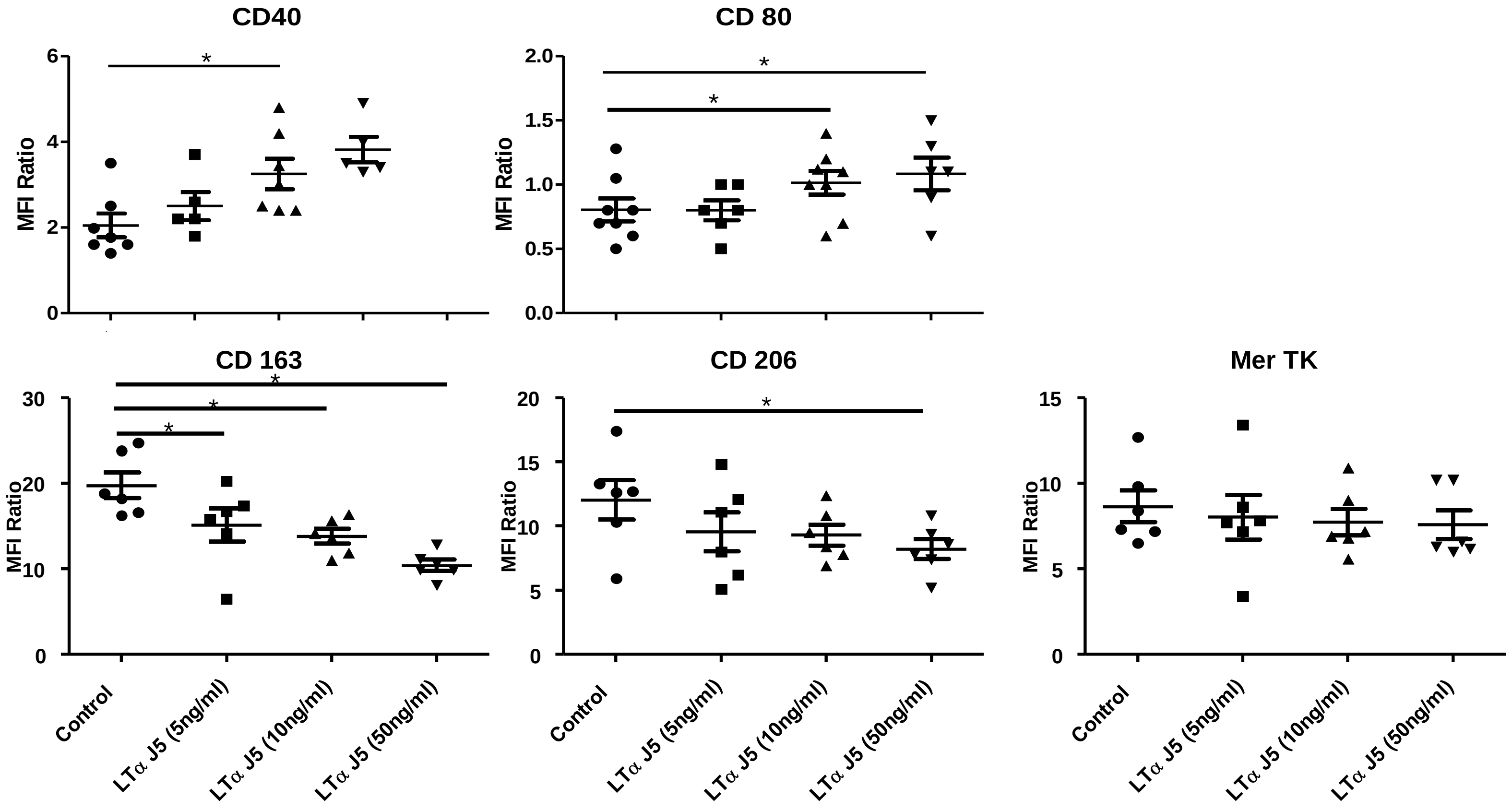

Monocytes were differentiated into macrophages in the presence of recombinant M-CSF (50 ng/ml) for 6 days, Human recombinant Lymphotoxin alpha (LTα) (5, 10 or 50 ng/ml) on day 5.
Ratio was calculated by dividing fluorescence of markers over fluorescence obtained with isotypes.

Supplementary Figure S2: Gating strategy and original histograms of monocytes labeling

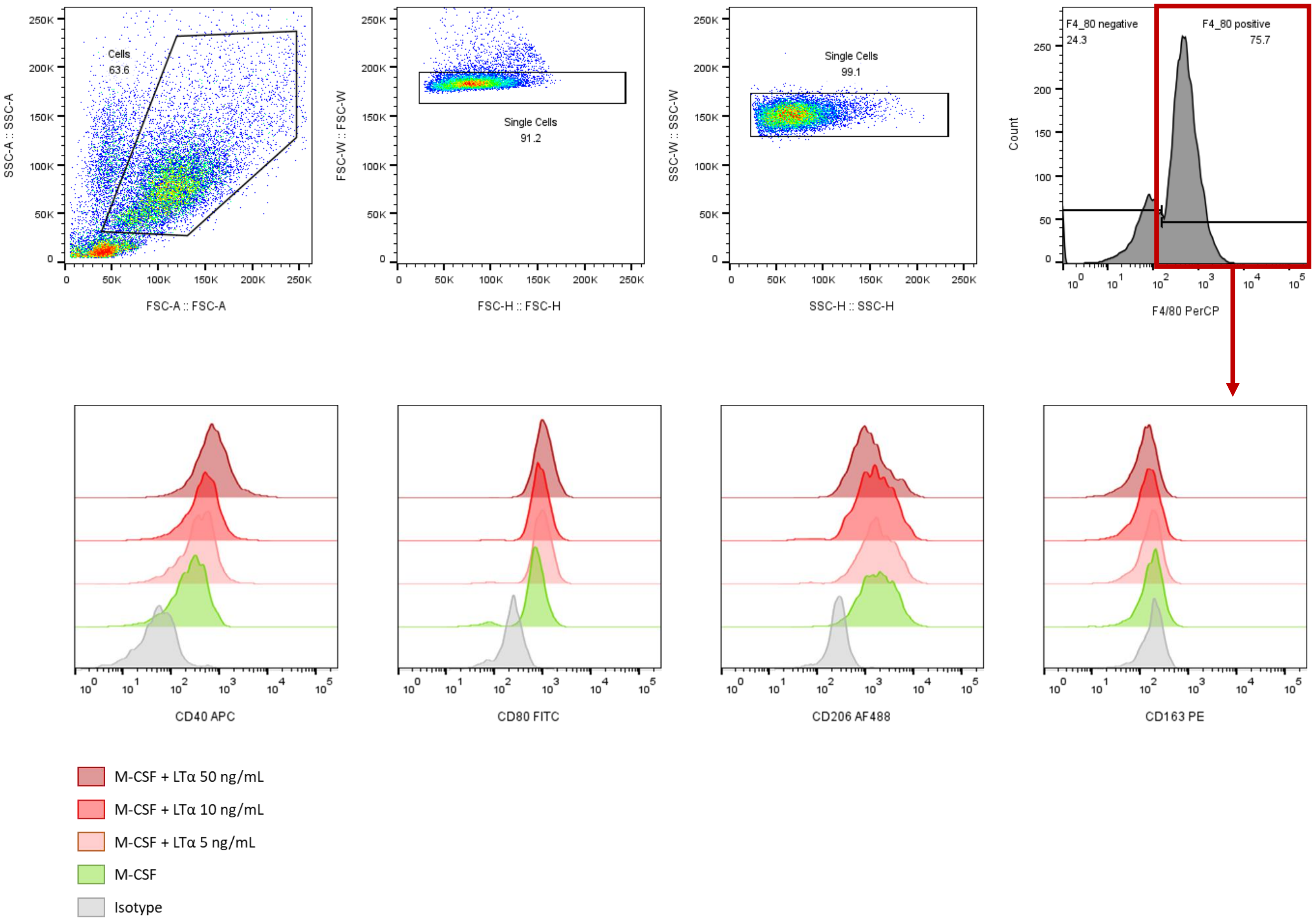

Supplement: Supplementary file 1 [file ijms-26-06355-s001.zip › ijms-3661340-supplementary.pdf]
